# The ultra-processed food content of school meals and packed lunches in the United Kingdom (2008-2017)

**DOI:** 10.1093/eurpub/ckac129.416

**Published:** 2022-10-25

**Authors:** JC Parnham, K Chang, F Rauber, RB Levy, S von Hinke, AA Laverty, C Millett, EP Vamos

**Affiliations:** 1 Public Health Policy Evaluation Unit, Imperial College London, London, UK; 2 Department of Nutrition, University of São Paulo, São Paulo, Brazil; 3 Department of Preventive Medicine, University of São Paulo, São Paulo, Brazil; 4 School of Economics, University of Bristol, Bristol, UK

## Abstract

**Background:**

British children have the highest ultra-processed food (UPF) intake in Europe, which is linked to adverse health outcomes. Schools are posited as a setting for dietary intervention, yet the level of UPFs consumed at schools is currently unknown. This study aimed to describe the UPF content of school food in the UK, explore the UPF content of school meals and packed lunches (food from home) and examine whether UPF differs by children's household income.

**Methods:**

A pooled cross-sectional analysis of primary (4-11 years, n = 1,895) and secondary schoolchildren (11-18 years, n = 1,408) from the UK's National Diet and Nutrition Survey (2008-2017) was conducted. Food diaries recorded student's meal-type (school meal/packed lunch). UPF intake was defined using the NOVA food classification system. Quantile regression models assessed the association between meal-type and lunchtime UPF intake (%kcal and % grams). Models were stratified by school phase (primary/secondary) and interacted meal-type with income.

**Results:**

Schoolchildren consumed most of their lunch as UPF, with higher median intakes in secondary schoolchildren than primary schoolchildren (77.8 %kcal vs 72.6 %kcal). School meals were associated with lower median UPF intake (%kcal) in both primary (-20 percentage-points[pp] [95% CI -22.2, -17.4]) and secondary schoolchildren (-11pp [-16.0,-7.0]) compared with packed lunches. Results were similar when UPF %g was analysed. Overall, income was inversely associated with UPF content. However, in primary schoolchildren there was no significant income gradient in the UPF(%g) content of school meals.

**Conclusions:**

In the first nationally representative study, we showed that on average UPF intake was high in all UK schoolchildren. Higher UPF intakes were observed in packed lunch consumers, secondary schoolchildren, and those with a lower income. Procurement policies must be revaluated to protect children from high UPF intake.

Funders: NIHR School for Public Health Research

**Key messages:**

• In the first study of ultra-processed food content of UK school food, we show that children consumed around three quarters of their energy as ultra-processed food at lunch.

• Children who were older, took food from home or were from a low-income household were more likely to consume higher levels of ultra-processed food. Regulation is needed to protect these children.

